# Structural mechanism of phospholipids translocation by MlaFEDB complex

**DOI:** 10.1038/s41422-020-00404-6

**Published:** 2020-09-03

**Authors:** Ximin Chi, Qiongxuan Fan, Yuanyuan Zhang, Ke Liang, Li Wan, Qiang Zhou, Yanyan Li

**Affiliations:** 1 Center for Infectious Disease Research, Zhejiang Provincial Laboratory of Life Sciences and Biomedicine, Key Laboratory of Structural Biology of Zhejiang Province, School of Life Sciences, Westlake University, Hangzhou, Zhejiang 310024 China; 2 Institute of Biology, Westlake Institute for Advanced Study, Hangzhou, Zhejiang 310024 China

**Keywords:** Electron microscopy, Membrane trafficking

## Abstract

In Gram-negative bacteria, phospholipids are major components of the inner membrane and the inner leaflet of the outer membrane, playing an essential role in forming the unique dual-membrane barrier to exclude the entry of most antibiotics. Understanding the mechanisms of phospholipid translocation between the inner and outer membrane represents one of the major challenges surrounding bacterial phospholipid homeostasis. The conserved MlaFEDB complex in the inner membrane functions as an ABC transporter to drive the translocation of phospholipids between the inner membrane and the periplasmic protein MlaC. However, the mechanism of phospholipid translocation remains elusive. Here we determined three cryo-EM structures of MlaFEDB from *Escherichia coli* in its nucleotide-free and ATP-bound conformations, and performed extensive functional studies to verify and extend our findings from structural analyses. Our work reveals unique structural features of the entire MlaFEDB complex, six well-resolved phospholipids in three distinct cavities, and large-scale conformational changes upon ATP binding. Together, these findings define the cycle of structural rearrangement of MlaFEDB in action, and suggest that MlaFEDB uses an extrusion mechanism to extract and release phospholipids through the central translocation cavity.

## Introduction

Gram-negative bacteria are distinctively characterized by their dual lipid membranes consisting of inner membrane (IM) and outer membrane (OM) that are separated by the aqueous periplasm and a layer of peptidoglycan. Both membrane leaflets of the IM are mostly composed of phospholipids, while the OM is an asymmetric lipid bilayer with the inner leaflet mainly composed of phospholipids and the outer leaflet of lipopolysaccharide (LPS). This asymmetric lipid bilayer serves as a permeation barrier, helps maintain optimal membrane fluidity, facilitates proper cellular function, and provides antibiotic resistance.^1,2^ Proteins involved in maintaining the asymmetrical OM are attractive targets for developing new classes of antibiotics to combat drug-resistant Gram-negative bacteria.

To build a stable and functional asymmetric OM, phospholipids and LPS must be transported across the periplasm from their site of synthesis, i.e., the cytoplasmic side of the IM. Previous studies demonstrate that LPS transport is powered by two ATP-binding cassette (ABC) transporters: MsbA and LptB_2_FGC.^3^ MsbA flips the nascent LPS across the IM.^4^ LptB_2_FGC extracts the mature LPS out of the IM,^5–8^ and subsequently pushes it to the other Lpt proteins in the periplasm and OM leading to LPS display on the cell surface.^9,10^ Compared to the considerable understanding achieved for the protein machinery involved in LPS transport, the mechanism and structural basis of phospholipid transport across the cell membranes remain unclear.

**Fig. 1:**
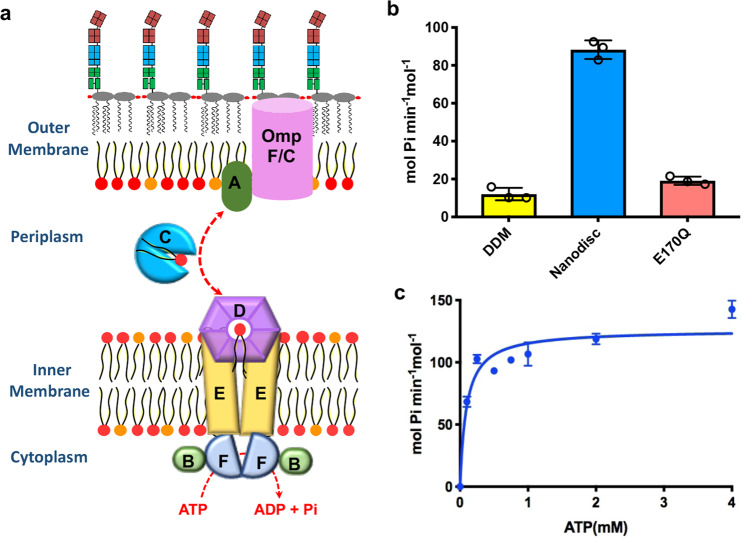
Biochemical characterization and cryo-EM studies of MlaFEDB. **a** Diagram of the Mla system for phospholipids transport. The Mla system proteins are labeled A through F. The inner membrane is composed of phospholipids, while the outer membrane is an asymmetric lipid bilayer with an inner leaflet composed of phospholipids and an outer leaflet of lipopolysaccharide. **b** ATPase activities of MlaFEDB complex in DDM and nanodisc, and MlaF_EQ_EDB in nanodisc. **c** Assay of the ATP concentration-dependent ATPase activities of nanodisc-embedded MlaFEDB complex. Each point represents mean ± SD. *n* = 3 biologically independent samples.

The maintenance of lipid asymmetry (Mla) proteins (Mla A-F) system is conserved in Gram-negative bacteria and plays an important role in phospholipid transport^11–13^ (Fig. 1a). This system consists of the IM transporter complex MlaFEDB, the periplasmic protein MlaC, and the OM lipoprotein MlaA. MlaA is found to form stable complexes with porins OmpF and OmpC and likely removes phospholipids from OM.^14,15^ MlaC has been crystallized with a phospholipid bound in a hydrophobic cavity, and functions as a periplasmic ferry between the IM and OM.^16,17^ The IM MlaFEDB complex was proposed to accept phospholipids from MlaC and insert them into the IM,^17,18^ suggesting a retrograde model. However, this idea was challenged by more recent observations. These studies demonstrate that the Mla system functions by transporting phospholipids in the opposite direction, starting from the IM and reaching MlaC through MlaD,^19,20^ suggesting a anterograde model. While the directionality of phospholipid movement induced by the Mla system is still under debate, it remains poorly defined at the mechanistic level how the Mla proteins drive the movement of phospholipids.

ATP-binding cassette (ABC) transporters are found in all organisms from bacteria to humans, harnessing the energy from ATP binding and hydrolysis to actively translocate a multitude of chemically diverse substrates across cell membranes.^21,22^ ABC transporters are comprised of two transmembrane domains (TMDs), which serve as specific substrate translocation pathway, and two nucleotide-binding domains (NBDs), which sandwich and hydrolyze ATP. The IM MlaFEDB complex functions as an ABC transporter to translocate phospholipids between the IM and periplasm (Fig. 1a). This complex is composed of four components, namely the NBD protein MlaF, the TMD protein MlaE, the phospholipid-binding protein MlaD, and the auxiliary protein MlaB. MlaE and MlaF, each forming a homodimer, interact with each other. MlaB was suggested to interact with and modulate the stability of MlaF.^18^ MlaD forms a hexameric disc-shaped ring on top of MlaE, with a central pore lined by hydrophobic residues and has been shown to contain phospholipids.^17,23^ Two low-resolution structures of MlaFEDB complex (8.7 and 10 Å) have provided the first glimpse into this complex.^17,19^ However, the details of the transporter complex assembly, the interactions with substrate phospholipids, and the transport mechanisms remain unclear.

In this study, we focus on the overall structural assembly, phospholipid interactions and conformational rearrangements of *Escherichia coli* (*E. coli*) MlaFEDB using single-particle cryo-EM. The structure of nucleotide-free MlaFEDB at 2.9 Å resolution shows the detailed interactions between individual protein components and bound phospholipids. The structures of ATP-bound complex reveal characteristic conformational rearrangements that couple ATP binding to phospholipid translocation. The closure of the inner cavities upon ATP binding suggests that MlaFEDB drives phospholipid movement using an extrusion mechanism. Thus, our results provide unprecedented structural insights into the phospholipid translocation pathway, uncover a previously unknown mechanism of phospholipid transport by the Mla system, and suggest potential molecular targets that could be exploited for antibiotic development.

## Results

### Biochemical and functional characterization of the MlaFEDB complex

In *E. coli*, the Mla system genes are located in an operon *mlaFEDCB*, which is conserved among Gram-negative bacteria. To understand the function of the complex, the full *mlaFEDCB* operon was overexpressed in *E. coli* C43(DE3), purified in dodecyl maltoside (DDM), and reconstituted in nanodisc with palmitoyl-oleoyl-phosphatidylglycerol (POPG) (Supplementary information, Fig. S1a–c). After the MlaFEDCB complex in nanodisc was purified, all protein components except MlaC were identified on SDS-PAGE, and MlaC was identified by mass spectrometry (Supplementary information, Fig. S2i–l). These results demonstrate that MlaFEDB can form a stable complex whereas binding of MlaC is likely to be transient.

The ATPase activity of MlaFEDB in nanodisc was substantially higher than the complex in DDM (Fig. 1b), indicating that lipid membrane is important for the function of the transporter. Mutating the catalytic glutamate residue to a glutamine (E170Q on MlaF) reduced the ATPase activity of MlaFEDB in nanodisc (Fig. 1b). Assay of the ATP concentration-dependent ATPase activities of MlaFEDB in nanodisc yielded a *K*_m_ of 0.09 ± 0.02 mM and a *V*_max_ of 126 ± 4.6 mol ATP per min per mol MlaFEDB (Fig. 1c).

The deletion of *mla* genes increases OM permeability, and results in growth defect with increased sensitivity to OM stressors such as SDS and EDTA.^12,24^ To understand how the *mla* genes function together in vivo to maintain the OM integrity, we constructed the *E. coli* strain with the whole operon *mlaFEDCB* deleted. The deletion strain showed growth defects in the presence of SDS and EDTA, which was rescued by complementation with MlaFEDCB (Supplementary information, Fig. S5).

### Overall structure of the MlaFEDB transporter complex

**Fig. 2:**
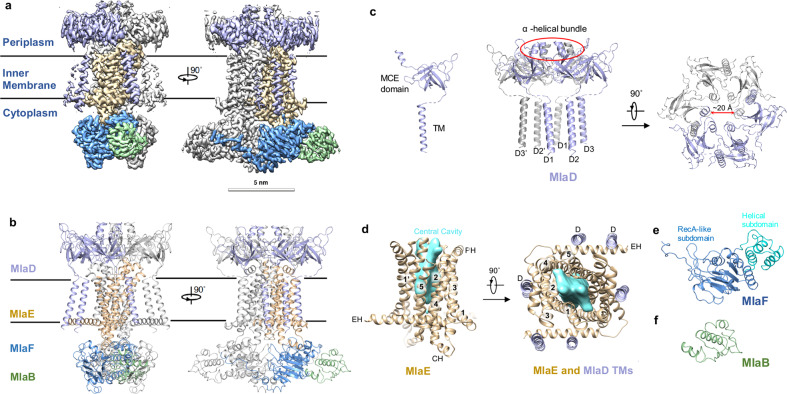
Overall structure of the MlaFEDB transporter complex. **a** Cryo-EM map of MlaFEDB. **b** Overall structure of the MlaFEDB transporter shown in cartoon. MlaD, MlaE, MlaF and MlaB are shown in light blue, wheat, actinium and pale green, respectively. **c** The cartoon structures of single subunit and hexameric MlaD. C-terminal α-helical bundle on top of the ring is shown. N-terminal TMs insert into the membrane. **d** The cartoon structures of dimer of MlaE (left), and the top view of the TMs of MlaE and MlaD (right). The surface cartoon of the hydrophobic cavity is shown in cyan. **e** The cartoon structure of MlaF. **f** The cartoon structure of MlaB.

To understand how the individual components are assembled together in the MlaFEDB complex, the structure of MlaFEDB in nucleotide-free form was determined by cryo-EM. The two-dimensional class averages of MlaFEDB showed clear structural features, suggesting high level of conformational homogeneity (Supplementary information, Fig. S4a). The final map of the MlaFEDB complex at 2.9 Å resolution reveals all side-chain densities and most secondary structural elements (Fig. 2a; Supplementary information, Fig. S4). MlaFEDB is assembled in a stoichiometry of 2:2:6:2 (MlaF:MlaE:MlaD:MlaB) (Fig. 2b). The minimum ABC transporter is composed of two copies of MlaE and MlaF serving as the TMDs and NBDs, respectively. Associated with the MlaF and MlaE homodimers are the unique auxiliary proteins: MlaB and MlaD. The two MlaB proteins in the cytoplasm do not contact each other, and each interacts with one MlaF.

**Fig. 3:**
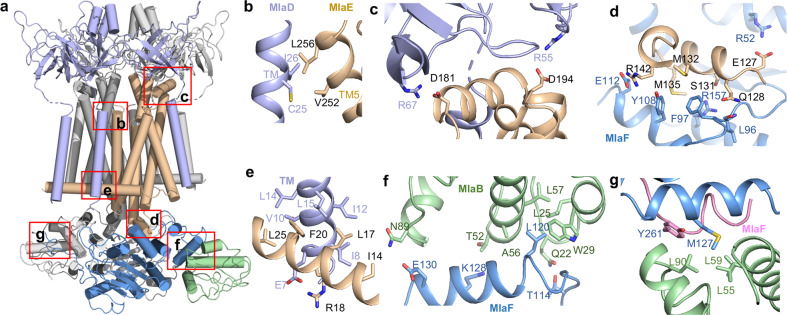
Interactions of individual subunits in MlaFEDB. **a** Overall cylindrical helix cartoon view of MlaFEDB, the interactions of individual subunits are boxed in red. **b** Zoomed-in view of the interaction between TM_D2_ of MlaD (light blue) and TM5 of MlaE (wheat). **c** Zoomed-in view of the interaction between MCE domain of MlaD (light blue) and the PH of MlaE (wheat). **d** Zoomed-in view of the interaction between the CH of MlaE (wheat) and MlaF (actinium). **e** Zoomed-in view of the interaction between TM_D2_ of MlaD (light blue) and the EH of MlaE (wheat). **f** Zoomed-in view of interaction of MlaB (pale green) and one MlaF subunit (actinium). **g** Zoomed-in view of interaction of MlaB′ (pale green) and two MlaF subunits (MlaF, pink; MlaF′, actinium).

MlaD contains a periplasmic mammalian cell entry (MCE) domain and a single N-terminal transmembrane helix (TM) (Fig. 2c, left). On the periplasmic side, the MCE domain of MlaD is assembled as a homo-hexameric ring with the C-terminal α-helical bundle on top of the ring (Fig. 2c, middle). The six α-helices form a hollow hydrophobic channel, presumably to allow for phospholipid transfer^17^ (Fig. 2c, right). The linker between the periplasmic MCE domain and TM was not well resolved, likely due to its flexible conformation. A total of six MlaD TMs in the MlaFEDB complex are inserted into the membrane (Fig. 2c, middle). Three MlaD subunits incorporate with one MlaE (Fig. 2c, middle, d, right). Transmembrane helices D1 and D2 of MlaD form close hydrophobic contacts with the elbow Helix (EH) of MlaE, and D3 contacts the TM1 and TM3 of MlaE (Figs. 2d, right, 3e). Our structure demonstrates that Ile14 in the EH of MlaE contacts Ile8 in the D2 of MlaD (Fig. 3e). The I14N mutation of MlaE indeed rendered the bacteria sensitive to the treatment of SDS/EDTA (Supplementary information, Fig. S5), suggesting that the interaction between the TM of MlaD and the EH of MlaE is critical for the phospholipid transport function of MlaFEDB.

MlaE forms a homodimer with each subunit containing one EH, five transmembrane helices (TM1–5), one coupling helix (CH) and one periplasmic helix (PH) (Fig. 2d, left). The EH, which runs parallel to the inner membrane plane, is similar to the connecting helix of human ABC transporters.^25,26^ However, EH is longer and not connected to the NBD. A central hydrophobic cavity is formed by the TM1, TM2, and TM5 of two subunits of MlaE (Fig. 2d). TM1 extends upwards toward the MlaE hydrophobic channel. The PH between TM3 and TM4 interacts with MlaD (Figs. 2d, left,  3c), and the CH connecting TM2 and TM3 contacts MlaF (Figs. 2d, left, 3d). The negatively charged residues D194 and D181 from the PH are accommodated by the positively charged residues R55 and R67 from MlaD (Fig. 3c). R142, S131, Q128, E127, M135 in CH of MlaE form close contacts with the E112, R157, L96, R52 and Y108 in MlaF, respectively (Fig. 3d).

The NBD domain of MlaF shows a conserved structure of known ABC transporters.^27,28^ Each MlaF contains a RecA-like subdomain, consisting of two β-sheets and five α-helices, and a helical subdomain formed by three α-helices (Fig. 2e). Notably, the C-terminus of one MlaF subunit extends across in a domain-swapped configuration to form close hydrophobic contacts with the opposing subunits MlaF′ and MlaB′ (Figs. 2a and 3g). The Y261 in MlaF and M127 in MlaF′ form hydrophobic interactions with L90, L59 and L55 in MlaB′. The M127A mutation of MlaF′ increased sensitivity of bacteria to SDS/EDTA, suggesting that the interaction in these three subunits is important for phospholipid transport (Supplementary information, Fig. S5).

MlaB in our structure represents the structurally characterized auxiliary protein that interacts with the NBD of ABC transporters. Two MlaB subunits form close interaction with MlaF and are located on the opposite side of the MlaF dimer (Figs. 2b and 3f). MlaB contains a STAS domain^29,30^ (Fig. 2f), which was reported to fuse to the transmembrane domain in transporters.^31–33^ Here in the Mla system, The T52 and Q22 in MlaB form close contacts with K128 and T114 in MlaF. The point mutations of MlaB (T52A and Q22A) in the interface caused increased sensitivity of bacteria to SDS/EDTA (Fig. 3f; Supplementary information, Fig. S5). The T52A mutation also abolished the ATPase activity of MlaFEDB.^18^ The results indicate that MlaB is critical for the assembly and the activity of the MlaFEDB complex.

### Phospholipids bound in and outside the MlaFEDB complex

To determine whether the MlaFEDB complex can bind phospholipids, purified MlaFEDB in DDM and nanodisc were subjected to Bligh–Dyer lipid purification and analyzed using mass spectrometry (Supplementary information, Fig. S2a–d); phosphatidylglycerol (PG) and phosphatidylethanolamine (PE) were detected and confirmed by using MS/MS (Supplementary information, Fig. S2e–h). Similar lipid binding was previously observed using thin-layer chromatography (TLC).^18^ These results demonstrate that phospholipids can be copurified with MlaFEDB, but the potential pockets for phospholipid interaction were not defined. Therefore, we further investigated the details of lipid binding by analyzing the MlaFEDB structures.

**Fig. 4:**
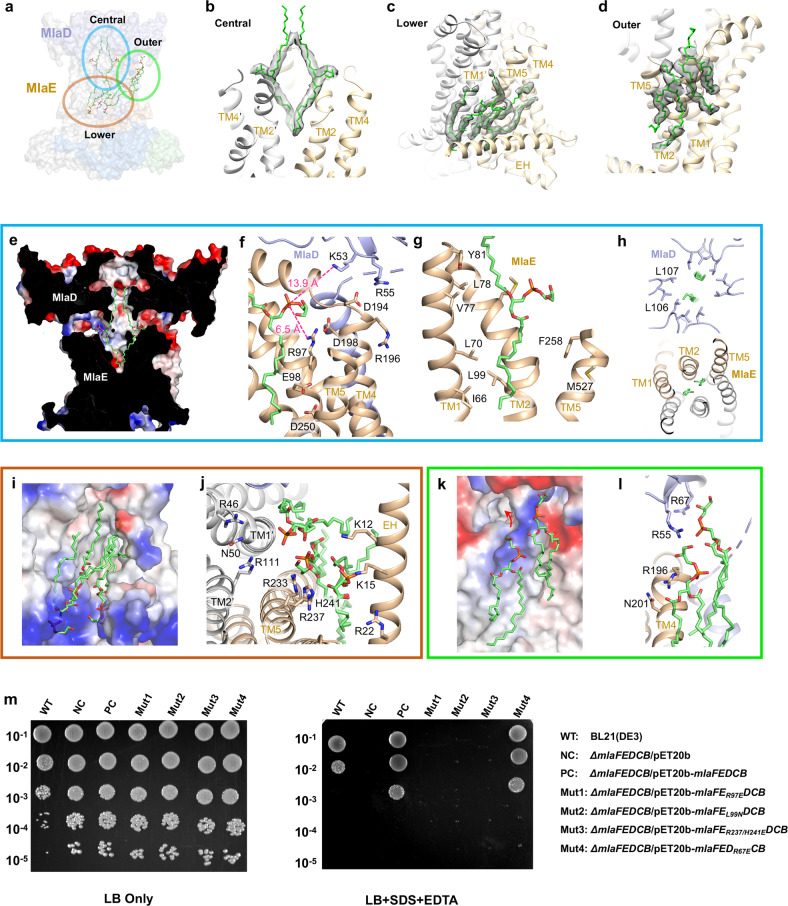
Interactions between phospholipids and MlaFEDB. **a** Overall view of all the phospholipids bound in the center, lower-side and outer-side cavities of MlaFEDB. The lipids bound in the cavity are circled in blue, orange and green, respectively. Zoomed-in views of the circled regions are shown in **b**–**d**, and the colored boxes in **e**–**l** correspond to the zoom-ins shown in **b**–**d**. **b**–**d** Zoom-in views of the phospholipid bound in the center, lower-side and outer-side cavities in MlaFEDB. Cryo-EM densities are shown superimposed with the atomic model of phospholipids. **e** Cross-sectional view of the electrostatic surface of the central cavity. **f**–**h** Zoomed-in view of detailed interactions between the phospholipids with MlaD and MlaE. Positively charged and negative charged regions are shown in blue and red, respectively. Phospholipids are shown as green sticks. MlaD and MlaE are shown in light blue and wheat, respectively. **i** Cross-sectional view of the electrostatic surface of the lower-side cavity. **j** Zoomed-in view of detailed interactions between the phospholipids and TMs of MlaD (gray) and MlaE (wheat). **k** Cross-sectional view of the electrostatic surface of the outer-side cavity. **l** Zoomed-in view of detailed interactions between the phospholipids with the MCE domain of MlaD (gray) and TM4 of MlaE (wheat). **m** Complementation of MlaFEDCB mutants in *MlaFEDCB* deletion strain.

Clearly defined diacyl phospholipids were observed to bind in the cavities of the MlaFEDB complex, and are located in three distinct regions formed by MlaE and MlaD (Fig. 4a), namely the central (Fig. 4b), lower (Fig. 4c) and outer cavities (Fig. 4d). All these phospholipid densities are well resolved showing the two acyl chains and the head group.

Two phospholipid molecules bound inside of the TMDs are located in the central hydrophobic cavity, which is formed by the TM1, TM2, and TM5 of two MlaE subunits and the hexameric ring of MlaD (Fig. 4b, e; Supplementary information, Fig. S6a). The phospholipid head group is positioned above the level of lipid membrane. The positively charged Lys53 and Arg55 in MlaD, together with the Arg97 and Arg196 in MlaE, form close contacts with the head group of the bound phospholipids (Fig. 4f). Consistent with the observed lipid-binding sites, the R97E mutation on the TM2 of MlaE increased sensitivity of bacteria to SDS/EDTA (Fig. 4m), suggesting the importance of this lipid-binding site for phospholipid transport. Notably, one acyl chain extends toward the MlaD and the other acyl chain extends deep into the hydrophobic cavity in MlaE, likely representing an intermediate state of the phospholipid being transported (Fig. 4e). The Leu106 and Leu107 in MlaD, and Leu99, Leu70, Val77, Leu78, Met89 and Ile66 in MlaE, form hydrophobic interactions with the acyl chains of the bound lipids (Fig. 4g, h). The isosteric mutations L106N/L107N in the hydrophobic cavity of MlaD failed to complement the *mlaD* deletion mutant as previously reported,^17^ which is consistent with a role of this cavity in MlaD function and phospholipid transport. L99N mutation on TM2 of MlaE reduced the bacterial growth and increased the sensitivity to SDS/EDTA (Fig. 4m), underscoring the functional importance of the hydrophobic cavity.

Three phospholipids were resolved in the lower cavity formed by the TM1′, TM2′, TM3′, TM4, TM5 and EH of MlaE and three MlaD TMs (Fig. 4c; Supplementary information, Fig. S6b). The head groups of the bound phospholipids interact with the positively charged residues (Fig. 4i) from the EH (Arg22, Lys12 and Lys15) and the TM5 of MlaE (Arg237 and Arg233); Arg111 and Arg46 from the opposing MlaE likely also contribute to the interaction (Fig. 4j). The R237E/H241E mutations on the TM5 of MlaE increased sensitivity of bacteria to SDS/EDTA (Fig. 4m), demonstrating that this lower lipid-binding cavity is critical for phospholipid translocation. Phospholipid binding in the lower cavity can contribute to the structural integrity of the MlaFEDB complex by stabilizing the encompassing protein elements, and may also have an active role in extracting lipid molecules from the surrounding cytoplasmic membrane leaflet.

Another two phospholipid molecules were observed in the outer cavity on the surface of MlaE (Fig. 4d; Supplementary information, Fig. S6c). The head groups of these lipids interact with the Arg55 and Arg67 of MlaD, and also with the Arg196 of MlaE (Fig. 4l). The acyl chains contact numerous hydrophobic residues of the TMs of MlaE and MlaD (Supplementary information, Fig. S6c, right). These two lipids are at the similar level of height to the phospholipids bound in the central cavity. The nearby TMs form a V-shaped open groove (Supplementary information, Fig. S6c, middle), which may represent a gateway for phospholipid entry and/or exit.

In summary, the three cavities in and outside the TMDs are specific and critical for phospholipid binding through both hydrophobic and electrostatic interactions. Our structure of MlaFEDB shows the interactions with side-chain details, providing the structural basis for understanding the phospholipid translocation pathway. The observation of phospholipids in the nucleotide-free structure also suggests that the phospholipids can enter the inner cavity independent of ATP. The ATP binding and hydrolysis are presumably coupled with the conformational changes to facilitate the phospholipid translocation.

### Structural rearrangement of MlaFEDB upon ATP binding

To reveal the conformational changes that enable the phospholipid translocation, we pursued the structure of MlaFEDB in the ATP-bound conformation. Dimerized MlaF subunits contain two active ATPase sites comprising the Walker A and Walker B motifs and the signature motif LSGG.^22^ The highly conserved glutamate residue on Walker B acts as the catalytic base for the ATP hydrolysis.^22^ Mutating the catalytic glutamate residue with a glutamine (E170Q on MlaF) severely reduced the ATPase activity of MlaFEDB by ~80% as compared to the wild-type protein (Fig. 1b). As expected, this mutation also induced a significant growth defect (Supplementary information, Fig. S5). For cryo-EM studies, the purified MlaFEDB complex with the E170Q mutation (MlaF_EQ_EDB) was incubated with 2 mM ATP-Mg^2+^ prior to the preparation of cryo-EM specimen. Image processing generated cryo-EM maps of the MlaF_EQ_EDB complex in different conformations (EQ_tall_ and EQ_close_) at resolutions of 4.3 and 3.7 Å, respectively (Supplementary information, Figs. S7b and S8a–h). EQ_close_ structure demonstrates tight closure of ATP-binding sites with the Walker A and signature motifs in close proximity, with a clear ATP-like density in between, which is consistent with an ATP-bound conformation. In contrast, the ATP-binding sites in EQ_tall_ structure are more open, suggesting that the two MlaF subunits do not adopt an ATP-bound conformation with tightly dimerized NBDs (Supplementary information, Fig. S8i). The maintaining of 20% ATPase activity suggests that the EQ mutant can bind and slowly hydrolyze ATP, and the EQ mutant was not fully effective in stabilizing the MlaFEDB in the NBD-closed conformation.

**Fig. 5:**
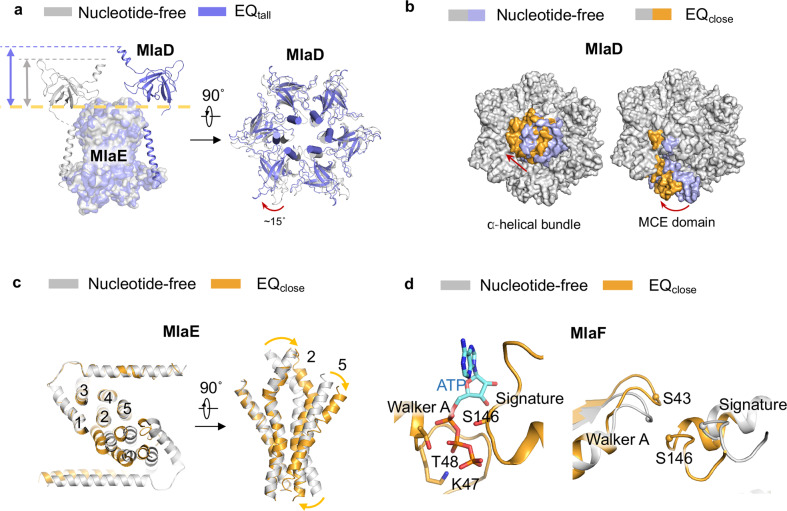
Structures of MlaF_EQ_EDB in the ATP-bound conformations. **a** Overlay of MlaD in the nucleotide-free and ATP-bound EQ_tall_ conformations. The MCE domain of MlaD exhibits upward movement compared with the nucleotide-free state, which is indicated by slate and gray arrows. **b** Overlay of MlaD in the nucleotide-free and ATP-bound EQ_close_ conformations. **c** Overlay of MlaE in the nucleotide-free and ATP-bound EQ_close_ conformations. Yellow arrow indicates the bending of TMD of MlaE. **d** Overlay of MlaF in the nucleotide-free and ATP-bound EQ_close_ conformations. ATP bound to the Walker A and signature motifs in EQ_close_ state is shown. T48, K47 and S146 participate in coordination with ATP mostly through its phosphate tail.

The structure of EQ_tall_ showed a major conformational rearrangement of MlaD. The hexametric ring of the MCE domain in MlaD is lifted, increasing the height of the entire complex (Fig. 5a, left). The ring of MCE domains also twisted clockwise, when viewed from the periplasmic side, by ~15° (Fig. 5a, right; Supplementary information, Video S1). The linker between the periplasmic ring and the TM of MlaD is flexible and remains invisible in the structure. The MlaE, MlaF and MlaB are essentially identical to those in the structure of nucleotide-free complex. The lift-up movement of MCE domain of MlaD leads to an extension of the central hydrophobic cavity formed by MlaE and MlaD to the periplasmic side, which may facilitate the movement of phospholipids to and/or from the periplasm. The density traces of a phospholipid tail was observed in the central cavity, suggesting the EQ_tall_ is in a transit state of lipid translocation (Supplementary information, Fig. S8j). Thus, our structure suggests that the binding of ATP results in conformational changes of the central translocation cavity and the phospholipid-binding protein MlaD.

The structure of EQ_close_ showed tight dimerization of the two MlaF subunits, caused by the trapping of ATP-Mg^2+^ at the ATP-binding sites (Fig. 5d; Supplementary information, Fig. S8i). The dimerization of the MlaF engages the CHs between TM2 and TM3 of MlaE, which induces the rearrangement of all TMs. The two TMDs move toward each other (Fig. 5c; Supplementary information, Video S2). TM1, TM2 and TM5 bend clockwise, when viewed from the front side, to collapse the central phospholipid-binding cavity (Fig. 5c), and accordingly no bound phospholipids were observed in the collapsed central cavity. The TMD conformational change further induce the movement of MlaD. The MlaD hexametric ring twisted and shifted around 30° as aligned to the nucleotide-free structure (Fig. 5b). No lift-up motion is observed. The EQ_close_ structure may represent an extrusion conformational change coupled with ATP binding to release and extract the bound phospholipids from the central cavity of MlaE.

## Discussion

The MlaFEDB complex was investigated as an ABC transporter to translocate phospholipids between the IM and OM of Gram-negative bacteria.^7,19,20^ Our cryo-EM studies of the MlaFEDB complex revealed the detailed information of the complex assembly. MlaD modulates the activity of ATPase via the interaction with MlaE. MlaD with the hexametric ring and hydrophobic groove allows the phospholipids to dissociate from the rest of the ABC transporter, and six IM-spanning TMs interact with TMD of MlaE to drive the conformation change. The MlaB is tightly associated with MlaF, and the C-terminus of one MlaF subunit extends across in a domain-swapped configuration to form close hydrophobic contacts with the opposing subunit of MlaF and MlaB. The high-quality cryo-EM map of the nucleotide-free MlaFEDB allows us to resolve the bound phospholipids and identify the critical binding determinants in side-chain details. The high-quality cryo-EM map of the nucleotide-free MlaFEDB also allows us to resolve the bound phospholipids inside the central cavity of MlaE in the outward-open conformation. This conformation shows two open NBDs, which suggests that ATP is not required for the substrate binding.

**Fig. 6:**
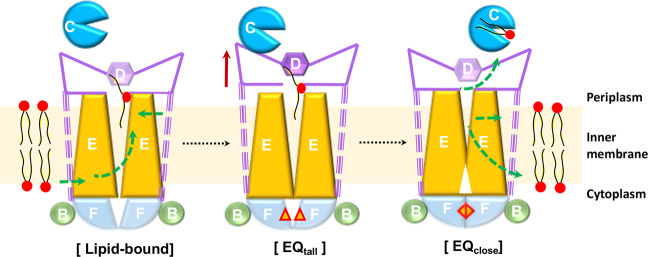
Proposed phospholipid extrusion mechanism mediated by MlaFEDB. The MlaE, MlaF and MlaB are shown in yellow, light blue and green, respectively. The nucleotide is indicated as red diamond. The green dash lines suggest the proposed phospholipid transfer direction.

The ATP-bound structures suggest that the MlaFEDB complex releases and extracts the bound phospholipids from the translocation cavity through an extrusion mechanism. The structure of EQ_tall_ suggests that MlaD lift-up may facilitate the transport of the phospholipids bound inside the central cavity, which may help to move the bound phospholipids upwards to reach MlaD in the periplasm. EQ_close_ structure elucidates that the ATP binding-coupled conformational change further collapses the central phospholipid-binding cavity and squeezes out the bound phospholipids (Fig. 6). However, it is not clear whether the bound phospholipids have been transferred to MlaC suggesting an anterograde model or to the membrane suggesting a retrograde model. Future structural and biochemical studies to determine the precise phospholipid-binding locations during the action of the Mla system are required to fully delineate the detailed mechanism of Mla-mediated phospholipid transport.

## Materials and methods

### Expression and purification of MlaFEDCB

The MlaFEDCB operon was amplified from *E. coli* K-12 genomic DNA by PCR. The fragment was digested with *Nde*I/*Xho*I and subsequently ligated into the pET20b plasmid. The recombinant plasmid pET20b-*mlaFEDCB* including an C-terminal His-tag on MlaB was used to transform *E. coli* C43(DE3) for expression. The bacterial cells were grown at 37 °C in Luria broth (LB) medium with 100 μg/mL Ampicillin until the optical density of the culture reached 1.0 at 600 nm. Protein expression was induced with 0.5 mM isopropyl β-D-1-thiogalactopyranoside (IPTG) and cells were grown for 12 h at 16 °C. Cells were harvested by centrifugation, flash frozen in liquid nitrogen and stored at −80 °C. Frozen cell pellets were re-suspended in lysis buffer containing 25 mM Tris, pH 7.8, 300 mM NaCl and 10% (v/v) glycerol and lysed by microfludizer. Unbroken cells and large debris were removed by centrifugation at 10,000 × *g* for 30 min at 4 °C. Membranes were pelleted by ultra-centrifugation at 100,000 × *g* for 1 h at 4 °C, re-suspended in lysis buffer, and solubilized with 1% (w/v) n-dodecyl-β-D-maltopyranoside (DDM, Anatrace) for 1 h at 4 °C. MlaFEDB were purified over TALON metal affinity resin (Clontech) followed by size-exclusion chromatography on a Superdex 200 column in a buffer containing 25 mM Tris, pH 7.8, 150 mM NaCl, 0.05% DDM and 5% glycerol. Protein fractions with the highest homogeneity were collected and concentrated to 6 mg/mL and stored at −80 °C. SDS-PAGE and mass spectrometry analysis revealed the presence of MlaFEDB, MlaC was not detected by SDS but detected by MS.

### Nanodisc reconstitution

1-palmitoyl-2-oleoyl-sn-glycero-3-phosphoglycerol (POPG) (Avanti Polar Lipids) was solubilized in chloroform, dried under argon gas to form a thin lipid film, and stored under vacuum overnight. The lipid film was hydrated and re-suspended at a concentration of 10 mM POPG in a buffer containing 25 mM Tris, pH 7.8, 150 mM NaCl and 100 mM sodium cholate. Mla complex proteins, MSP1D1 membrane scaffold protein, and POPG were mixed at a molar ratio of 0.5:1:60 in a buffer containing 25 mM Tris, pH 7.8, 150 mM NaCl and 15 mM sodium cholate, and incubated for 1 h at 4 °C. Detergents were removed by incubation with 0.6 mg/mL Bio-Beads SM2 (Bio-Rad) overnight at 4 °C. Nanodisc-embedded MlaFEDB was purified using a Superdex 200 column in a buffer containing 25 mM Tris, pH 7.8, and 150 mM NaCl.

### ATPase assay

All ATPase activity assays were performed using ATPase/GTPase activity assay kit (Sigma). Briefly, 0.5 μg of purified Mla complex in nanodiscs were incubated in a 40 μL reaction volume containing 0–4 mM ATP for 30 min at 37 °C. The absorbance at 620 nm was measured. ATPase activities of all samples were determined using the mean value of the samples according to the linear regression of standards.

### Lipids purification and mass spectrometry

The lipids in these samples were purified by Bligh–Dyer isolation. The protein samples were converted to one phase solution of buffer/chloroform/methanol (0.8:1:2, v/v/v). After being incubated at room temperature for 30 min, the suspension was converted into a two-phase Bligh–Dyer system buffer/chloroform/methanol (1.8:2:2, v/v/v) by addition of buffer and methanol. After mixing thoroughly and centrifugation at 3000 × *g* for 5 min, the lower phase was collected and used for mass spectrometry analysis. Accurate mass measurements were acquired using an Agilent 6545 QTOF mass spectrometer. The parameters used for the mass spectrometer with ESI-negative mode were as follows: capillary voltage of 2.5 kV, nozzle voltage of 1000 V, gas temperature of 320 °C, drying gas of 8 L/min, nebulizer pressure of 35 psi, sheath gas temperature of 350 °C, sheath gas of 11 L/min. The mass spectrometer was operated both in MS mode and MS/MS mode, of which the collision energy is 20 or 30 eV. Scan range was from *m/z* 100 to 1000.

### Site-directed mutagenesis and complementation functional assays

All site-directed mutations were generated following the protocol of NEB Q5 site-directed mutagenesis kit. The mutants on pET20b-MlaFEDCB were transformed into the *E. coli MlaFEDCB*-null strain, plated on LB plates with 100 μg/mL Ampicillin and grown for 12 h at 37 °C. The wild-type pET20b-MlaFEDCB and empty vector pET20b were transformed into the *E. coli MlaFEDCB*-null strain and used as the positive and negative control, respectively. Single colonies were picked and inoculated into 5 mL LB supplemented with 100 μg/mL Ampicillin. The cells were harvested and then diluted in sterile LB to reach OD_600_ at 0.5. Each sample was subsequently serially diluted and the range was from 10^−1^ to 10^−6^. 1 μL of the diluted samples was spotted on LB plates containing 100 μg/mL Ampicillin and 0.5 mM IPTG with or without 0.8% SDS and 2.5 mM EDTA. The plates were incubated at 37 °C overnight and observed.

### Construction of *mlaFEDCB* deletion mutant

The *mlaFEDCB* deletion in the chromosome of *E. coli* was constructed. The upstream and downstream fragments of *mla* operon was amplified and overlapped together by PCR, resulted in the replacement fragment Ptrc-*mla*. The pTargetF-*mla* was obtained from pTargetF by inverse PCR and self-ligation. The plasmid pCas was transformed into *E. coli*. Arabinose was added to the culture when preparing the competent *E. coli*/pCas cells to activate the Red enzymes for recombination. 200 ng pTargetF-*mla* and 500 ng Ptrc-*mla* were electroporated into the *E. coli*/pCas competent cells. Cells were recovered at 30 °C for 2 h, then spread onto LB agar plates containing kanamycin and spectinomycin, and incubated for 48 h at 30 °C. The right colonies were confirmed by DNA sequencing. pTargetF-*mla* was cured by adding IPTG, and pCas was cured by growing at 42 °C overnight.

### EM sample preparation and data acquisition

To prepare samples for cryo-EM analysis, 3 μL of purified nanodisc-embedded MlaFEDB at a concentration of 4 mg/mL was applied to glow-discharged holey carbon grids (Quantifoil Au R1.2/1.3). For EQ mutant, the samples were incubated in a buffer containing 2 mM ATP and 2 mM MgCl_2_ for 30 min at the room temperature before applying the samples to cryo-EM grids. Grids were blotted for 4 s and plunge-frozen in liquid ethane cooled by liquid nitrogen using a Vitrobot Mark IV (Thermo Fisher). The prepared grids were transferred to Titan Krios electron microscopy operating at 300 kV equipped with Gatan K3 detector and GIF Quantum energy filter. The movie stacks were automatically collected using AutoEMation^34^ with slit width of 20 eV on the energy filter in super-resolution mode at nominal magnification of 81,000×. The defocus range was from −2.2 to −1.2 µm. Each stack included 32 frames with an exposure time of 0.08 s for each frame and a total dose rate of about 50 e^−^/Å^2^. The stacks were motion corrected MotionCor2^35^ and binned 2-fold, resulting in a pixel size of 1.087 Å/pixel. At the same time, dose weighting^36^ was operated. The defocus values were estimated by Gctf.^37^

### Data processing

For nucleotide-free MlaFEDB, RELION-3^38^ was used to automatically pick particles from manually selected micrographs. After 2D classification, good particles were selected and 3D classified against an initial model generated by RELION-3 with C2 symmetry. Good particles were selected and subjected to further 3D classification, local defocus correction (Gctf),^37^ 3D auto-refinement and post-processing. In order to further improve the map quality, adaptive masks were applied to the soluble region of MlaD with C6 symmetry and the soluble region of MlaFEB and the TM region with C2 symmetry, respectively. For ATP-bound MlaF_EQ_EDB, the particle picking and 2D classification were similar to that for nucleotide-free MlaFEDB. Good particles selected from 2D classification were 3D classified into six classes with C1 symmetry. Good classes were subjected to 3D classification focused on whole structure. To improve the map quality of vanadate-trapped MlaFEDB, the dataset was further focused on the intracellular region of MlaFEB and the TM region with C1 symmetry. Then the good classes were subjected to 3D auto-refinement and post-processing individually. The 2D classification, 3D classification and auto-refinement were performed with RELION 3. The resolution was estimated by using the gold-standard Fourier shell correlation of 0.143 criterion^39,40^ with high-resolution noise substitution.^41^ Details of data collection and processing are listed in Supplementary information, Figs. S3, S7b and Table S1.

### Model building and refinement

Model building of nucleotide-free MlaFEDB was performed ab initio with PHENIX^42^ and COOT^43^ based on the focused-refined cryo-EM maps with the aromatic residues used as markers, as most of these residues were clearly visible in cryo-EM map. During model building, each residue was manually checked with its chemical properties considered. Several segments of the complex were not modeled due to the invisibility of the corresponding density. For different conformations of MlaF_EQ_EDB bound with ATP, the atomic models were first generated by molecular dynamics flexible fitting (MDFF)^44^ of the nucleotide-free MlaFEDB model into the corresponding cryo-EM map. And then these models were further manually refined and checked in COOT. PHENIX is used to refine structure with secondary structure and geometry restraints to prevent structure overfitting. In order to monitor the overfitting of the model, the model was refined against one of the two independent half maps according to the gold-standard 3D refinement method. The improved model was tested against the other map.^45^ Statistics related to data collection, 3D reconstruction and model building is shown in Supplementary information, Table S1.

## Supplementary information

Supplementary information Figure S1

Supplementary information Figure S2

Supplementary information Figure S3

Supplementary information Figure S4

Supplementary information Figure S5

Supplementary information Figure S6

Supplementary information Figure S7

Supplementary information Figure S8

Supplementary information Table S1

Supplementary information, Video legend

Supplementary information, Video S1

Supplementary information, Video S2

## Data Availability

Three three-dimensional cryo-EM density maps of *E. coli* MlaFEDB in nanodiscs have been deposited in the Electron Microscopy Data Bank under accession numbers EMD-30355 (nucleotide-free MlaFEDB), EMD-30358 (EQ_tall_ state of MlaFEDB) and EMD-30367 (EQ_close_ state of MlaFEDB). Three atomic coordinates for the atomic models have been deposited in the Protein Data Bank under accession numbers 7CGE (nucleotide-free MlaFEDB), 7CGN (EQ_tall_ state of MlaFEDB), and 7CH0 (EQ_close_ state of MlaFEDB).
