# Supplementary material for: Structural mechanism of phospholipids translocation by MlaFEDB complex
Source: Cell Res. 2020 Sep 3;30(12):1127–35. doi: 10.1038/s41422-020-00404-6 (PMC7784689; doi:10.1038/s41422-020-00404-6)
Supplement: Supplementary file 1 — Supplementary information Figure S1 [file 41422_2020_404_MOESM1_ESM.pdf]

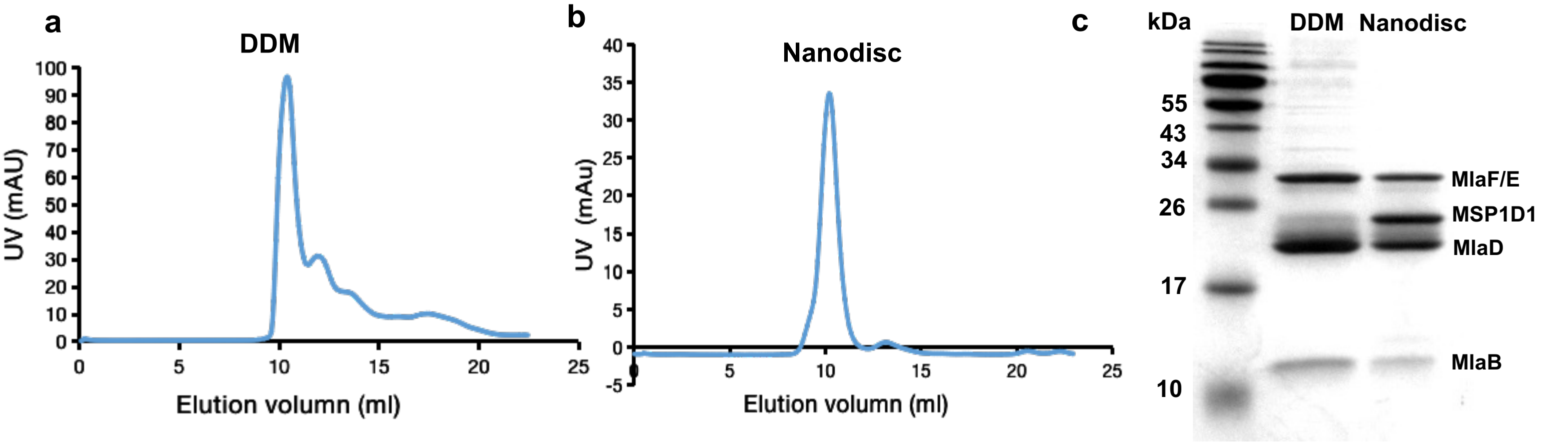

Supplementary information, Fig. S1 Purification and functional characterization of MlaFEDB in DDM and in nanodiscs. a, Gel-filtration chromatography profile of MlaFEDB in DDM. **b**, Gel-filtration chromatography profile of MlaFEDB in nanodiscs. **c**, Coomassie blue-stained SDS-PAGE gel of purified MlaFEDB in DDM and in nanodiscs. Individual protein components of the complex are labeled.
