# Supplementary material for: Structural mechanism of phospholipids translocation by MlaFEDB complex
Source: Cell Res. 2020 Sep 3;30(12):1127–35. doi: 10.1038/s41422-020-00404-6 (PMC7784689; doi:10.1038/s41422-020-00404-6)
Supplement: Supplementary file 2 — Supplementary information Figure S2 [file 41422_2020_404_MOESM2_ESM.pdf]

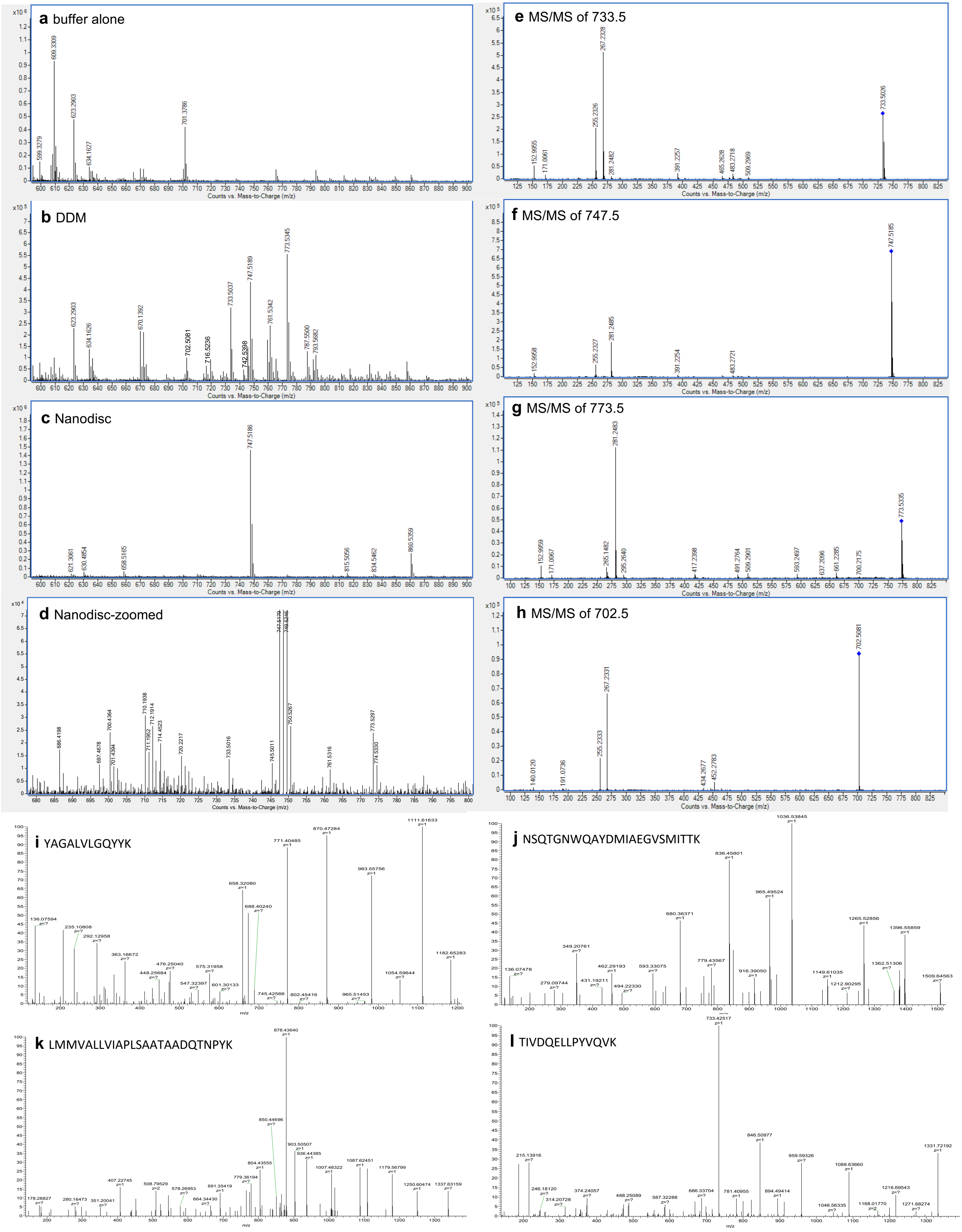
a buffer alone
b DDM
c Nanodisc
d Nanodisc-zoomed
e MS/MS of 733.5
f MS/MS of 747.5
g MS/MS of 773.5
h MS/MS of 702.5
Counts vs. Mass-to-Charge (m/z)
i YAGALVLGQYYK
j NSQTGNWQAYDMIAEGVSMITTK
k LMMVALLVIAPLSAATAADQTNPYK
l TIVDQELLPYVQVK
m/z

Supplementary information, Fig. S2 Mass spectrometry analysis of lipids isolated from MlaFEDCB in DDM and in nanodiscs. **a**, Mass spectra of lipids purified from buffer alone. **b**, Mass spectra of lipids isolated from purified MlaFEDCB in DDM, the ions at *m/z* 733.5, 747.5, 761.5, 773.5, 787.5 were identified as PG. The ions at *m/z* 702.5, 716.5 and 742.5 were identified as PE. **c**, Mass spectra of lipids purified from MlaFEDCB in POPG nanodisc. **d**, Mass spectra of lipids purified from MlaFEDCB in POPG nanodisc at zoomed range from *m/z* 680 to 800. **e**, Tandem MS spectra of the fragments at *m/z* 733.5 from purified MlaFEDCB in DDM. The fragment at *m/z* 733.5 is consistent with PG with two acyl chain of C16:0 and C17:1. **f** Tandem MS spectra of the fragments at *m/z* 747.5 from purified MlaFEDCB in DDM. The fragment at *m/z* 747.5 is consistent with PG with two acyl chain of C16:0 and C18:1. **g**, Tandem MS spectra of the fragments at *m/z* 773.5 from purified MlaFEDCB in DDM. The fragment at *m/z* 773.5 is consistent with PG with two acyl chain of C18:1 and C18:1. **h**, Tandem MS spectra of the fragments at *m/z* 702.5 from purified MlaFEDCB in DDM. The fragment at *m/z* 702.5 is consistent with PE with two acyl chain of C16:0 and C17:1. **i**, Tandem MS spectra of the fragments of YAGALVLGQYYK from MlaC in MlaFEDCB in nanodisc. **j**, Tandem MS spectra of the fragments of NSQTGNWQAYDMIAEGVSMITTK from MlaC in MlaFEDCB in nanodisc. **k**, Tandem MS spectra of the fragments of LMMVALLVIAPLSAATAADQTNPYK from MlaC in MlaFEDCB in nanodisc. **l**, Tandem MS spectra of the fragments of TIVDQELLPYVQVK from MlaC in MlaFEDCB in nanodisc.
