# Supplementary material for: Structural mechanism of phospholipids translocation by MlaFEDB complex
Source: Cell Res. 2020 Sep 3;30(12):1127–35. doi: 10.1038/s41422-020-00404-6 (PMC7784689; doi:10.1038/s41422-020-00404-6)
Supplement: Supplementary file 3 — Supplementary information Figure S3 [file 41422_2020_404_MOESM3_ESM.pdf]

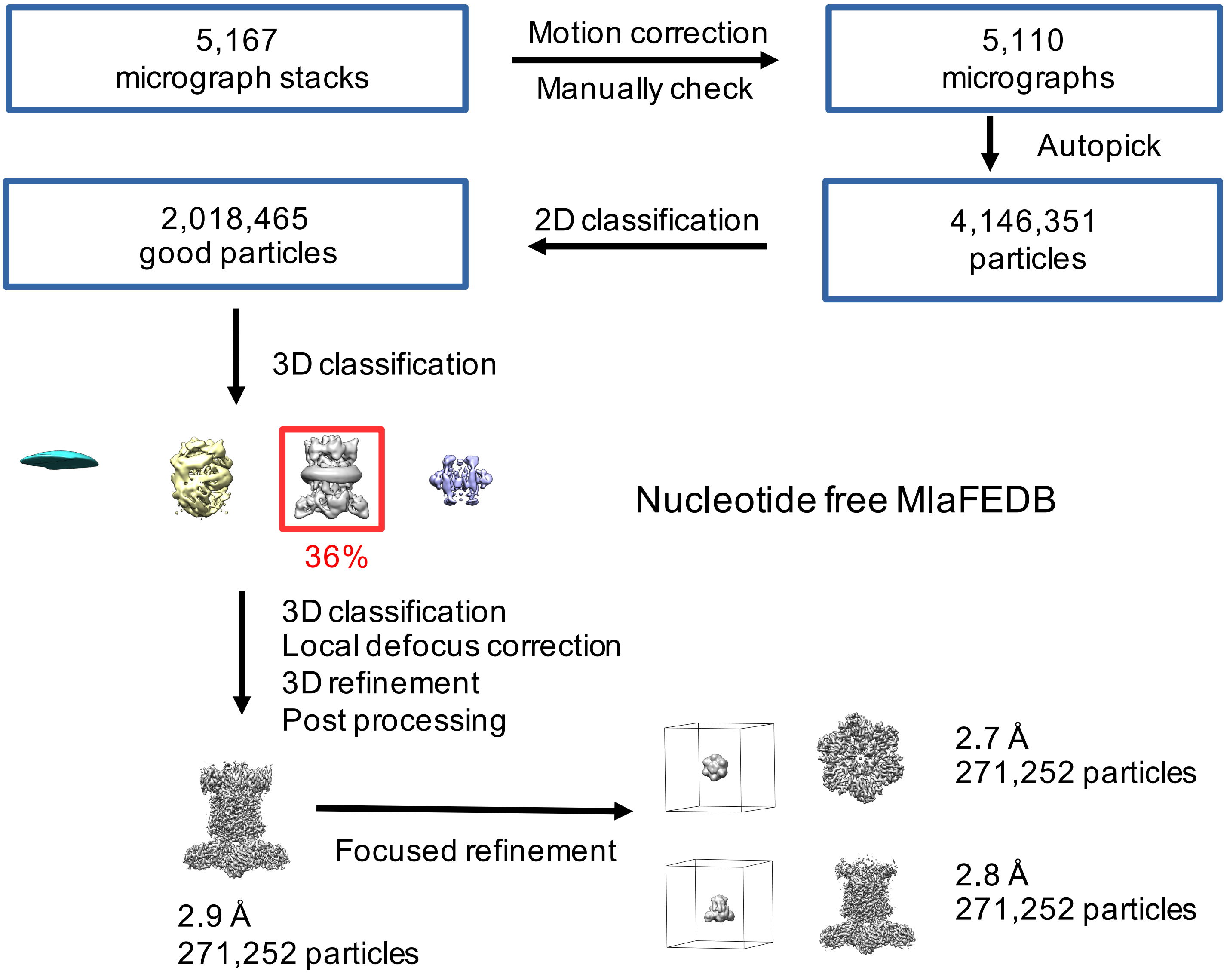

Supplementary information, Fig. S3 Image processing for the cryo-EM data of nucleotide-free MlaFEDB. Flow chart for cryo-EM data processing. For details, see 'Data processing' in the Methods section.
