# Supplementary material for: Structural mechanism of phospholipids translocation by MlaFEDB complex
Source: Cell Res. 2020 Sep 3;30(12):1127–35. doi: 10.1038/s41422-020-00404-6 (PMC7784689; doi:10.1038/s41422-020-00404-6)
Supplement: Supplementary file 4 — Supplementary information Figure S4 [file 41422_2020_404_MOESM4_ESM.pdf]

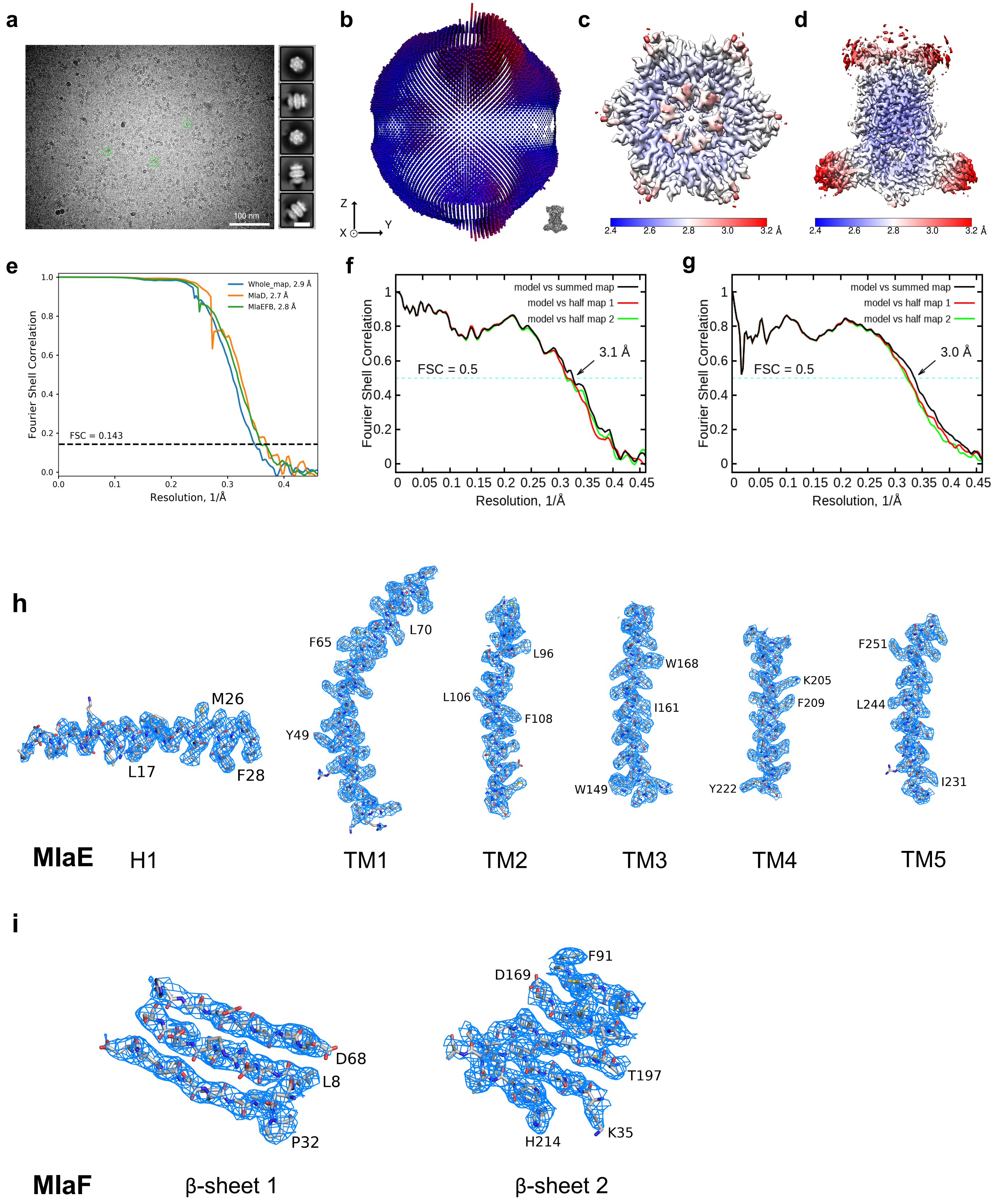

a
100 nm
b
Z
X
Y
c
2.4
2.6
2.8
3.0
3.2 Å
d
2.4
2.6
2.8
3.0
3.2 Å
e
Fourier Shell Correlation
Whole_map, 2.9 Å
MlaD, 2.7 Å
MlaEFB, 2.8 Å
FSC = 0.143
Resolution, 1/Å
f
Fourier Shell Correlation
model vs summed map
model vs half map 1
model vs half map 2
3.1 Å
FSC = 0.5
Resolution, 1/Å
g
Fourier Shell Correlation
model vs summed map
model vs half map 1
model vs half map 2
3.0 Å
FSC = 0.5
Resolution, 1/Å
h
M26
L17
F28
L70
F65
Y49
L96
L106
F108
W168
I161
W149
K205
F209
Y222
F251
L244
I231
MlaE
H1
TM1
TM2
TM3
TM4
TM5
i
D68
L8
P32
F91
D169
T197
H214
K35
MlaF
β-sheet 1
β-sheet 2

Supplementary information, Fig. S4 Single-particle cryo-EM analysis of nucleotide-free MlaFEDB in nanodiscs. **a**, Representative cryo-EM image and 2D class averages of cryo-EM particle images of nucleotide-free MlaFEDB in nanodiscs. **b**, Angular distribution of particle images included in the final 3D reconstruction. **c** and **d**, Local resolution of the final cryo-EM map of nucleotide-free MlaFEDB focused on the MlaD subunits or MlaFEB subunits, respectively. **e**, Fourier shell correlation (FSC) curves: gold-standard FSC curve for overall structure (blue), MlaD subunits (orange) and MlaFEB subunits (green) with indicated resolution at FSC=0.143, **f** and **g**, FSC curve of MlaD subunits (orange) and MlaFEB subunits between the atomic model and the final map with indicated resolution at FSC=0.5 (black); FSC curve between half map 1 (red) or half map 2 (green) and the atomic model refined against half map 1. **h**, Cryo-EM densities superimposed with the atomic model for individual TM helices in the nucleotide-free MlaE. **i**, Cryo-EM densities superimposed with the atomic model for selected regions of the NBDs (MlaF), demonstrating the clear separation of the β-strands and side chain densities.
