# Supplementary material for: Structural mechanism of phospholipids translocation by MlaFEDB complex
Source: Cell Res. 2020 Sep 3;30(12):1127–35. doi: 10.1038/s41422-020-00404-6 (PMC7784689; doi:10.1038/s41422-020-00404-6)
Supplement: Supplementary file 6 — Supplementary information Figure S6 [file 41422_2020_404_MOESM6_ESM.pdf]

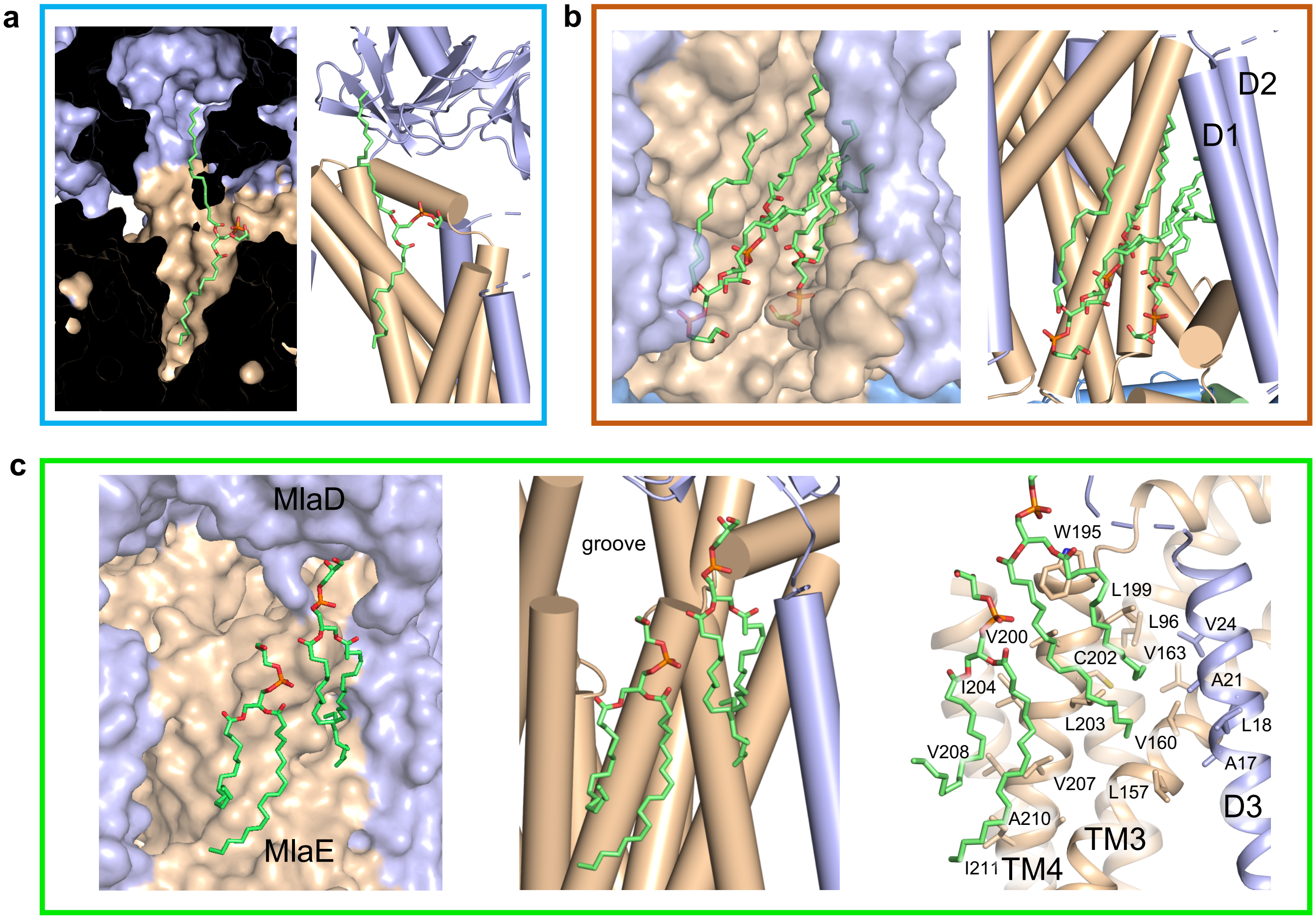

Supplementary information, Fig. S6 Interactions between phospholipids and nucleotides with MlaFEDB. a, Cross-sectional view of the phospholipids bound in the central cavity. MlaD and MlaE are shown in light blue and wheat, respectively. **b**, Cross-sectional view of the phospholipids bound in the lower side cavity. **c**, Cross-sectional view of the phospholipids bound in the upper side cavity, showing the tight fit of the phospholipid acyl chains into the hydrophobic cavity.
