# Supplementary material for: Structural mechanism of phospholipids translocation by MlaFEDB complex
Source: Cell Res. 2020 Sep 3;30(12):1127–35. doi: 10.1038/s41422-020-00404-6 (PMC7784689; doi:10.1038/s41422-020-00404-6)
Supplement: Supplementary file 7 — Supplementary information Figure S7 [file 41422_2020_404_MOESM7_ESM.pdf]

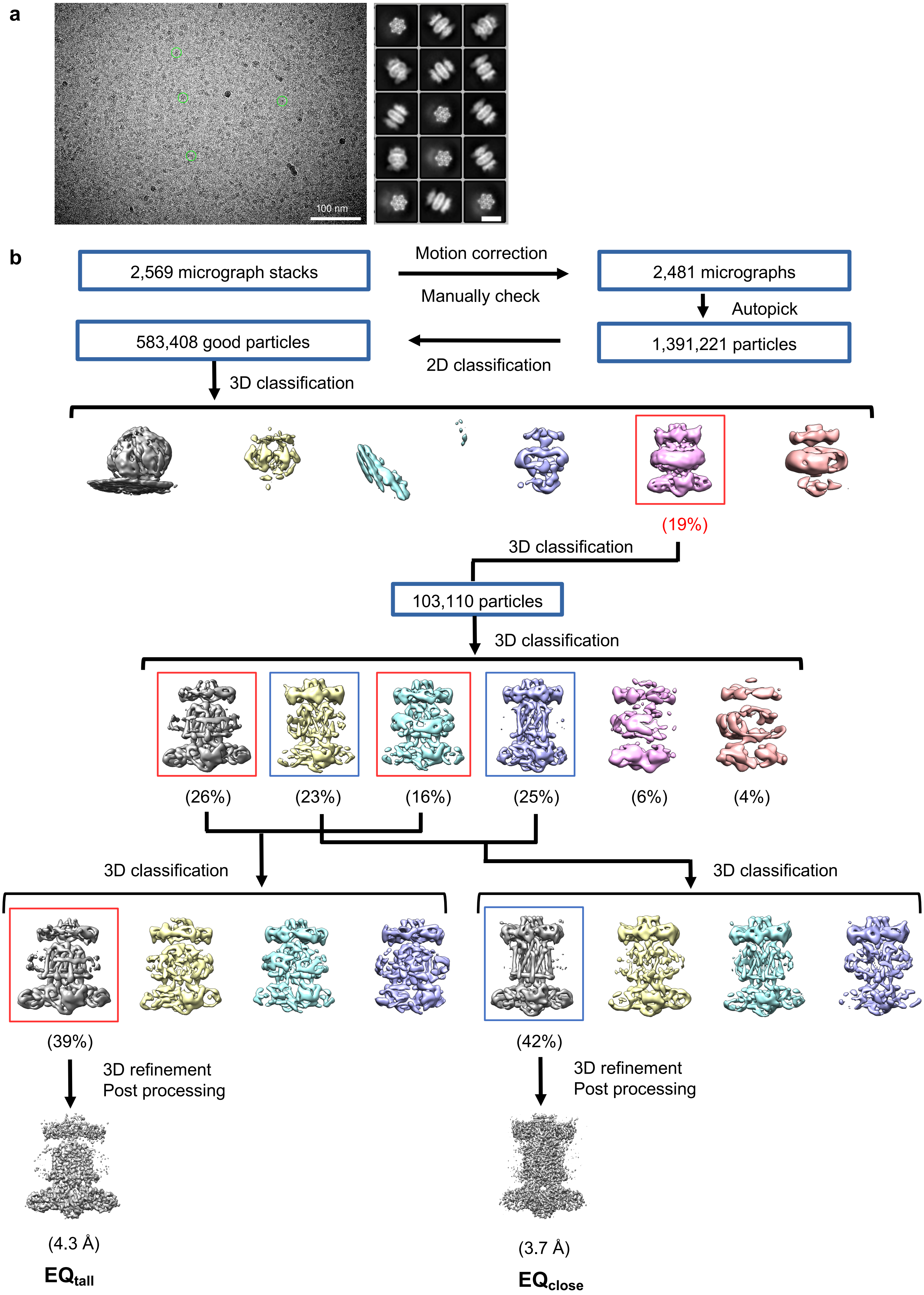
a
100 nm
b
2,569 micrograph stacks
Motion correction
Manually check
2,481 micrographs
Autopick
1,391,221 particles
2D classification
583,408 good particles
3D classification
(19%)
3D classification
103,110 particles
3D classification
(26%)
(23%)
(16%)
(25%)
(6%)
(4%)
3D classification
3D classification
(39%)
(42%)
3D refinement
Post processing
3D refinement
Post processing
(4.3 Å)
EQtall
(3.7 Å)
EQclose

Supplementary information, Fig. S7 Image processing for the cryo-EM data of ATP-bound MlaF$_{EQ}$EDB in nanodiscs. a, Representative cryo-EM image and 2D class averages of cryo-EM particle images of ATP-bound MlaF$_{EQ}$EDB in nanodiscs. **b,** Flow chart for cryo-EM data processing. For details, see 'Data processing' in the Methods section.
