# Supplementary material for: Structural mechanism of phospholipids translocation by MlaFEDB complex
Source: Cell Res. 2020 Sep 3;30(12):1127–35. doi: 10.1038/s41422-020-00404-6 (PMC7784689; doi:10.1038/s41422-020-00404-6)
Supplement: Supplementary file 8 — Supplementary information Figure S8 [file 41422_2020_404_MOESM8_ESM.pdf]

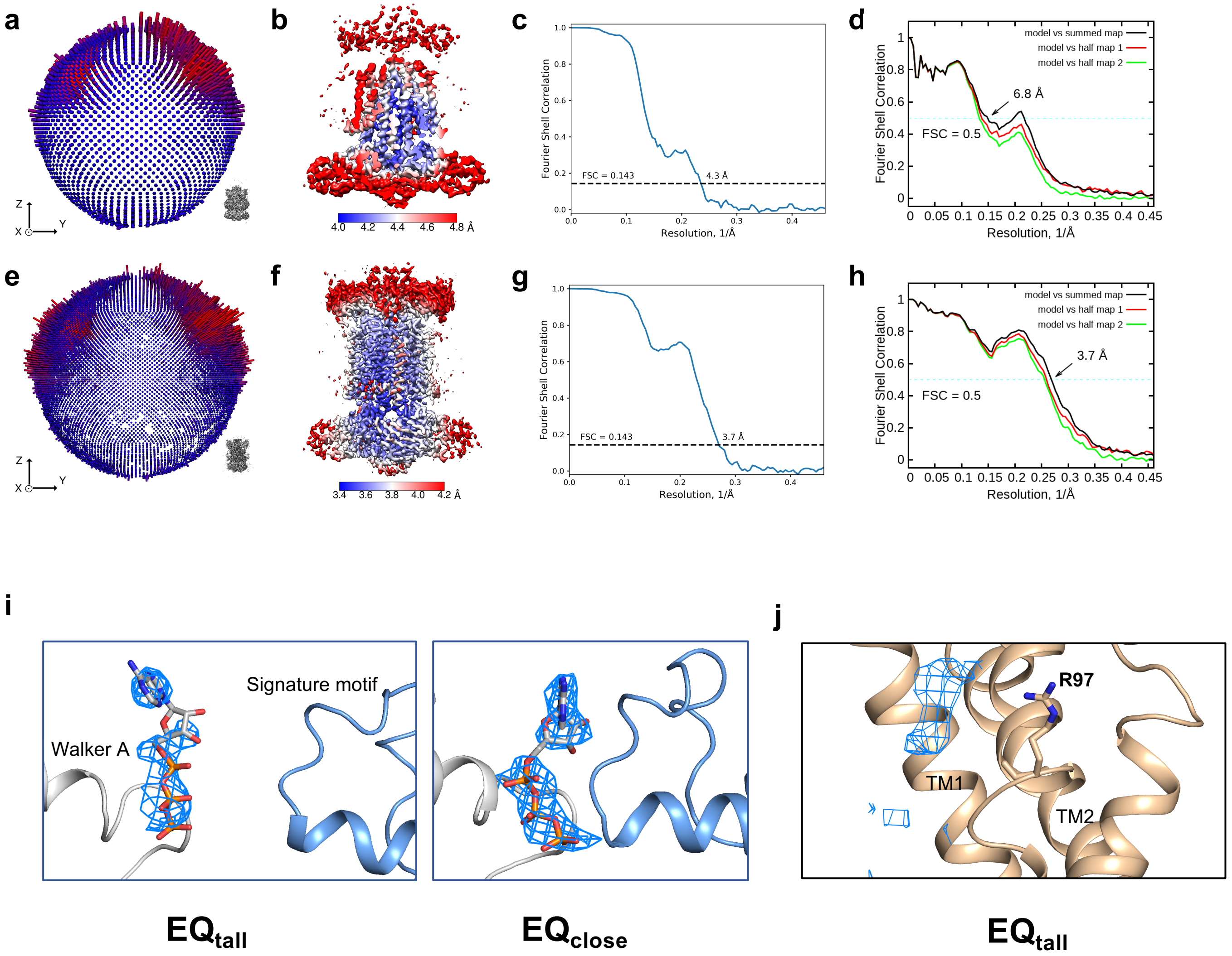

Supplementary information, Fig. S8 Single-particle cryo-EM analysis of ATP-bound MlaF$_{EQ}$EDB in nanodiscs. a-d cryo-EM analysis of EQ$_{tall}$ state. **a**, Angular distribution of particle images in EQ$_{tall}$ state. b, Local resolution of the final cryo-EM map of EQ$_{tall}$ state. **c**, Gold standard FSC curve for the 3D refinement of EQ$_{tall}$ state (blue). d, FSC curve of EQ$_{tall}$ state between the atomic model and the final map with indicated resolution at FSC=0.5 (black); FSC curve between half map 1 (red) or half map 2 (green) and the atomic model refined against half map 1. **e-h** are cryo-EM analysis of EQ$_{close}$ state, which are same to **a-d** in order. **i**, Cryo-EM densities for the nucleotides bound at the ATP-binding sites of two subunits of MlaF in EQ$_{tall}$ and EQ$_{close.}$ **j**, the interaction between the phospholipids and EQ$_{tall}$ state, the acyl chain density is shown in blue.
