# Supplementary material for: Structural mechanism of phospholipids translocation by MlaFEDB complex
Source: Cell Res. 2020 Sep 3;30(12):1127–35. doi: 10.1038/s41422-020-00404-6 (PMC7784689; doi:10.1038/s41422-020-00404-6)
Supplement: Supplementary file 9 — Supplementary information Table S1 [file 41422_2020_404_MOESM9_ESM.pdf]

Supplementary information, Table S1 Statistics of the cryo-EM structures presented in this study.

**Data collection**			
EM equipment	Titan Krios (Thermo Fisher Scientific)		
Voltage (kV)	300		
Detector	Gatan K3 Summit		
Energy filter	Gatan GIF Quantum, 20 eV slit		
Pixel size (Å)	1.087		
Electron dose (e-/Å2)	50		
Defocus range (μm)	-1.2 ~ -2.2		
Sample	Nucleotide-free MlaFEDB	ATP-bond $MlaF_{EQ}EDB$	
Number of collected micrographs	5,167	2,569	
Number of selected micrographs	5,110	2,481	
**3D Reconstruction**			
Software	Relion 3.0		
Comformation	Apo	EQ_{tall}	EQ_{close}
Number of used particles (Overall)	271,252	17,109	21,159
Resolution (Å)	2.9/ 2.7/ 2.8[a]	4.3	3.7
Symmetry	C2/ C6/ C2[a]	C1	
Map sharpening B-factor (Å2)	200/ 90/ 90[a]	150	
**Refinement**			
Software	Phenix		
Cell dimensions			
a=b=c (Å)	347.84	217.40	
α=β=γ (˚)	90	90	
Model composition			
Protein residues	2,092	2,092	2,092
Side chains assigned	2,038	2,038	2,038
lipid	12	N/A	N/A
ADP-vanadate	N/A	N/A	N/A
ATP	N/A	2	2
R.m.s deviations			
Bonds length (Å)	0.006	0.007	0.007
Bonds Angle (˚)	1.131	1.085	1.118
Ramachandran plot statistics (%)			
Preferred	87.74	88.96	87.84
Allowed	11.09	10.46	11.72
Outlier	1.17	0.58	0.44
