# Supplementary material for: Structural mechanism of phospholipids translocation by MlaFEDB complex
Source: Cell Res. 2020 Sep 3;30(12):1127–35. doi: 10.1038/s41422-020-00404-6 (PMC7784689; doi:10.1038/s41422-020-00404-6)
Supplement: Supplementary file 10 — Supplementary information, Video legend [file 41422_2020_404_MOESM10_ESM.pdf]

Supplementary information, Video legend

Supplementary information, Video S1 Conformational changes of MlaFEDB from nucleotide-free to ATP-bound EQ_{tall} state.

Supplementary information, Video S2 Conformational changes of MlaFEDB from nucleotide-free to ATP-bound EQ_{close} state.
